# Primary vitreous cysts

**DOI:** 10.3205/oc000145

**Published:** 2020-04-02

**Authors:** Pieter Robben, Rita Van Ginderdeuren, Daphne Thoma, Catherine Deghislage, Joachim Van Calster, Johan Blanckaert, Ingele Casteels

**Affiliations:** 1 Ophthalmology Department, University Hospitals Leuven, Belgium; 2 Ophthalmology Department, East Limburg Hospital, Genk, Belgium; 3 Ophthalmology Department, Jan Yperman Hospital, Ypres, Belgium

## Abstract

**Objective:** To report two cases of vitreous cysts with discussion of their pathophysiology and management.

**Methods:** Clinical examination with fundus photography, ultrasound and optical coherence tomography. Histopathology was performed in the first case.

**Results:** The first case illustrates a pigmented, free-floating cyst, which was removed during a 27-gauge vitrectomy. The histopathology shows a single layer of pigmented epithelium and confirms the previously reported presence of a PAS-positive basement membrane. The second case shows a sessile, non-pigmented cyst associated with significant anisometropia.

**Conclusion:** Primary vitreous cysts are rare and can have a wide range in their clinical aspect. This likely reflects whether they originate either from the pigment epithelium or the primary hyaloidal system. The management of vitreous cysts is mostly conservative, but pars plana vitrectomy can be used safely if the symptoms are debilitating.

## Introduction

Vitreous cysts were first described in 1899 by Tansley [1]; only a few cases were reported thereafter [2]. Vitreous cysts are classified as primary (idiopathic) and acquired, caused by trauma, degeneration, inflammation or infection. The origin of primary cysts remains debated, since histological evidence is scarce. To our knowledge only five reports of primary cysts with histopathological analysis were published [2], [3], [4], [5], [6]. Here, two cases of primary cysts, one with histopathological analysis, are reported.

## Case description

### Case 1

**Figure 1:**
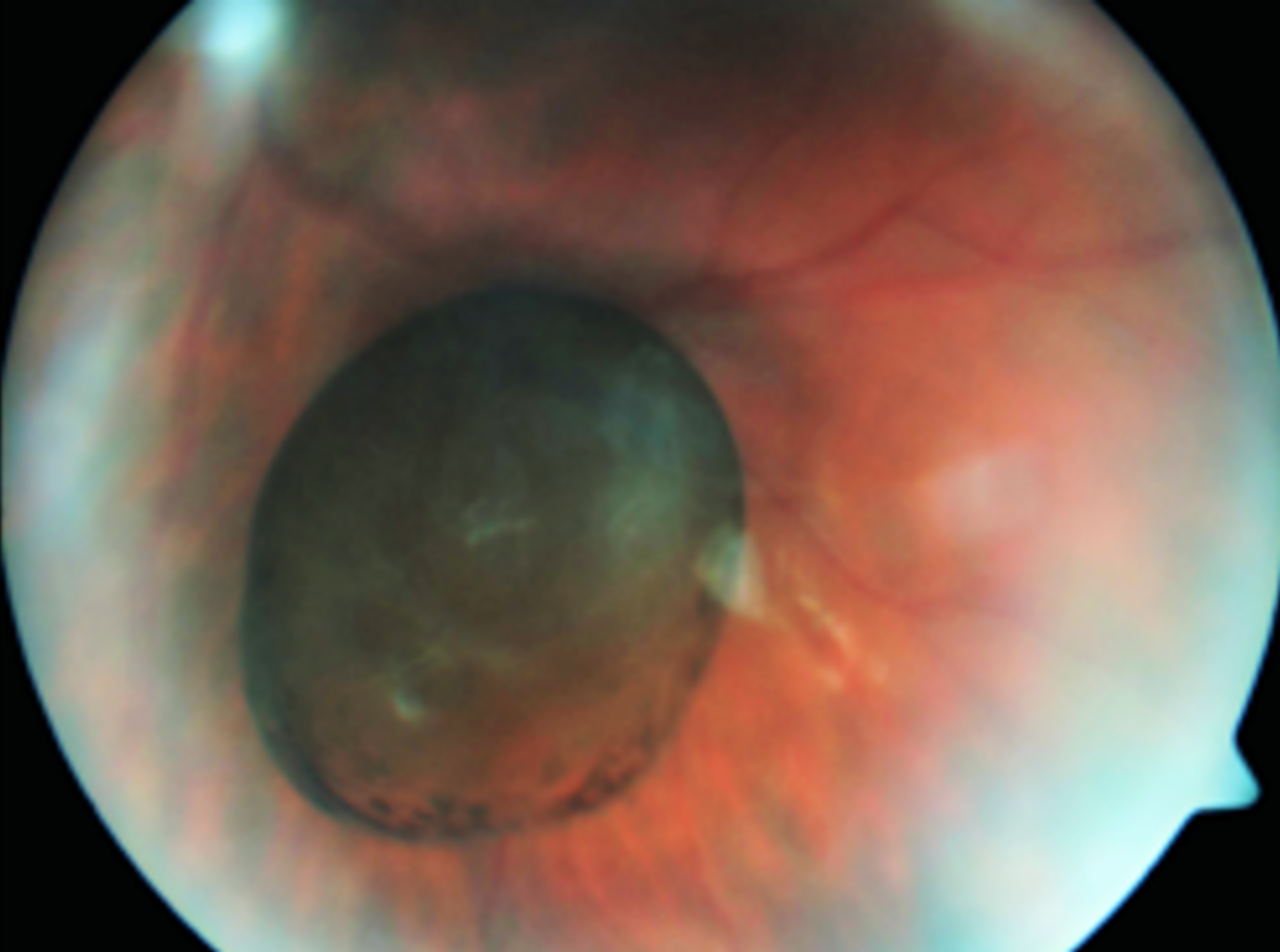
Fundus photograph showing the free-floating, pigmented vitreous cyst

A 15-year-old girl presented at East Limburg Hospital, Genk, with complaints of a transient scotoma on her left eye for four years. She was in good general health and there was no history of ocular trauma or infection. Her vision was LogMar 0.0 on the right and 0.5 on the left. Fundoscopy showed an oval, pigmented, translucent cyst in the left eye with no apparent attachments (Figure 1 (Fig. 1)). Further fundus examination was normal. Biomicroscopy was normal. There were no signs of intraocular inflammation. The patient’s refraction was +0.5 for the right eye and +0.75 for the left eye. Retinal OCT imaging was normal, but the cyst obscured the image intermittently. Ultrasound B-scan showed a hyperechogenic, spherical cyst, 4.1 mm in diameter. A-scan showed a low internal reflectivity. No attachments or calcifications were noted. The diameter of the cyst had measured 2.8 mm four years earlier, as described by Missotten et al. [7]. The cyst was aspirated during a 27-gauge vitrectomy, and the cyst wall was extracted and histologically examined. Postoperatively, the patient regained a vision of LogMar 0.0. A subtle posterior subcapsular cataract was seen, and subjectively some complaints of floaters were still reported.

**Figure 2:**
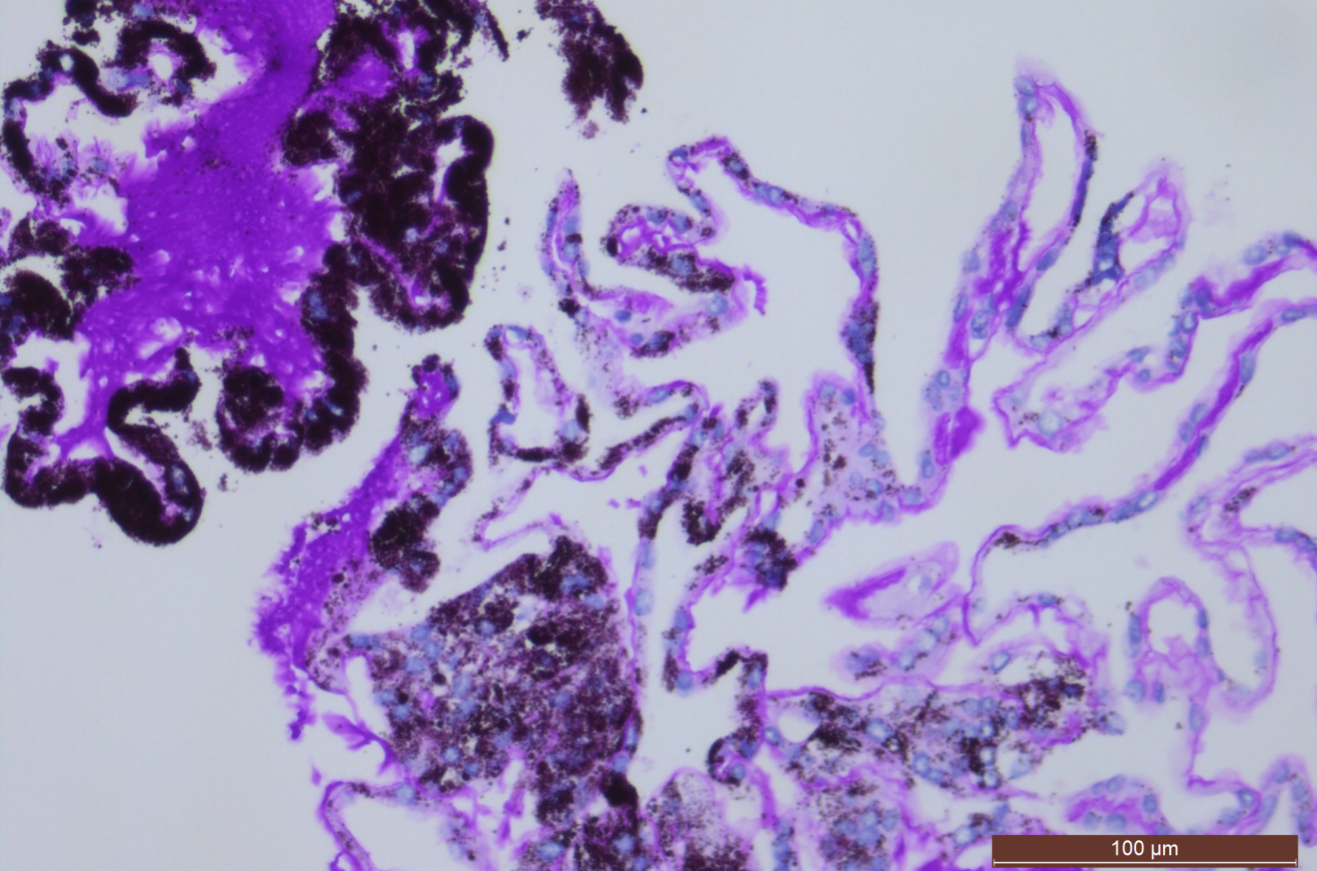
The single layer of cuboidal cells contain a variable amount of pigmented granules and are lined with a PAS-positive basement membrane (periodic acid-Schiff staining x20).

The aspirated vitreous sample was divided over four ThinPrep^®^ (Hologic Inc., Marlborough, MA, USA) containers, with Preservcyt^®^ (Hologic Inc., Marlborough, MA, USA) as fixative. Further handling was performed by the Cellient^®^ (Hologic Inc., Marlborough, MA, USA), an automated cell block system. Eight paraffin blocks were prepared according to the routine protocol for vitreous samples [8]. A collapsed cyst consisting of a single layer of partially pigmented cuboidal cells was seen on microscopy (Figure 2 (Fig. 2)). The cytoplasm had a variable amount of large black pigmented granules. There was no stratification, atypia, pleiomorphism or inflammation of the epithelium detected. A periodic acid-Schiff-positive basal membrane was visible on one side of the cells (Figure 2 (Fig. 2)). All the cells stained positive for the Pankeratin marker AE1/AE3, and were negative for the proliferation marker Mib1.

### Case 2

**Figure 3:**
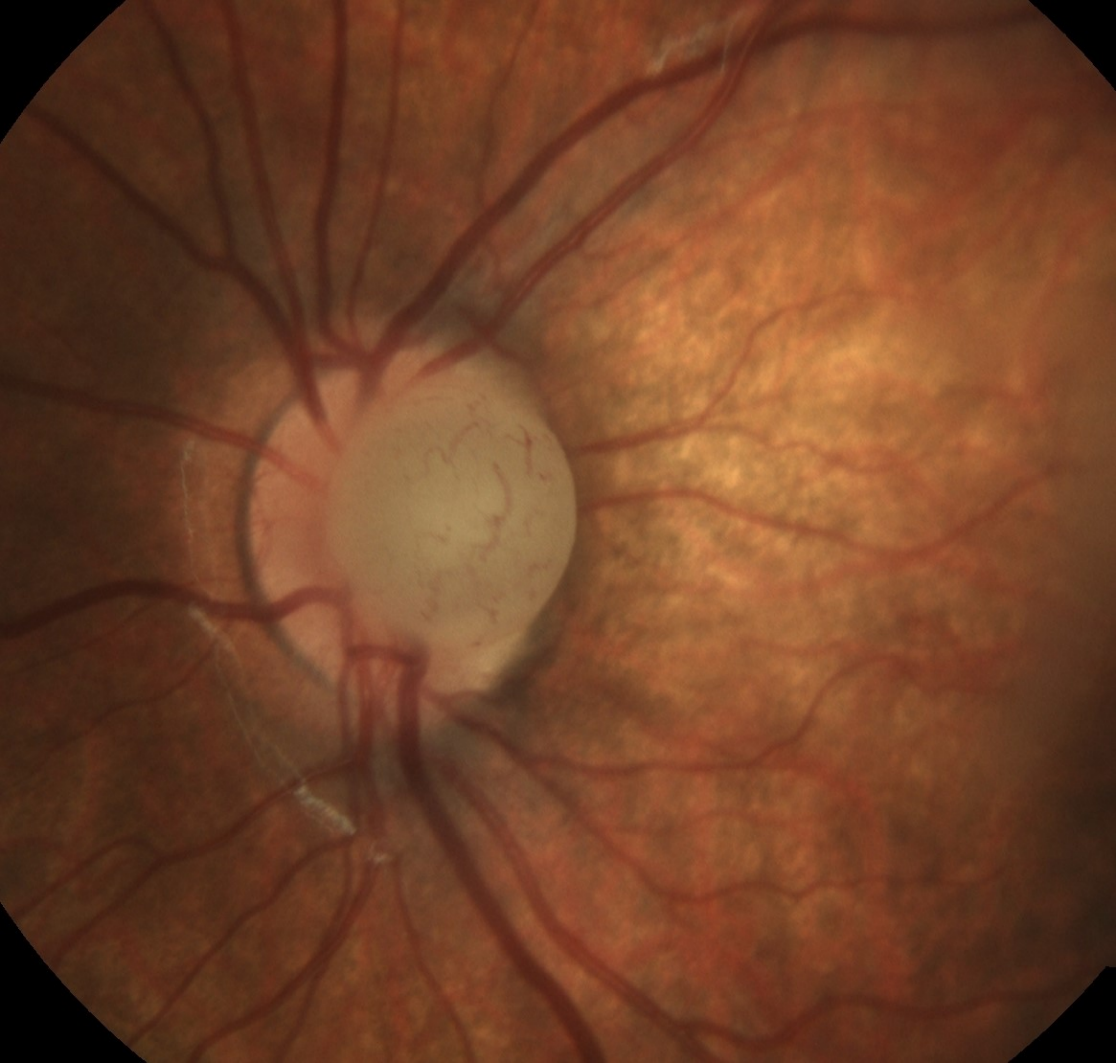
Fundus photograph showing the prepapillary cyst

A 15-month-old baby was referred to the ophthalmology service of the University Hospitals Leuven after a screening test perfomed by the children’s healthcare service had turned out abnormal. She had no special medical history. Pregnancy, childbirth and development were normal. Fundoscopy showed a round, whitish, vascularised prepapillary structure without transillumination on the left side, and was normal on the right side (Figure 3 (Fig. 3)). Biomicroscopy and eye pressure were normal. Ultrasound B-scan showed an attachment to the optic nerve. There was an anisometropia on cycloplegic refraction with +2.5 (–0.75x15°) for the right eye and –6 (–1x110°) for the left. A blood sample showed no signs of inflammation or eosinophilia. Spectacles were prescribed and patching of the right eye was initiated. At the age of three, the vision was LogMar 0.075 on the right and 0.725 on the left, measured with crowded Kay Pictures. During the follow-up, no change in size or aspect of the cyst was noted.

## Discussion

The embryologic origin of primary vitreous cysts remains controversial, due to both their low prevalence and the scarcity of histological studies. There are two main hypotheses about the origin of these cysts. The association of premelanosomes with the possible remnants of the pri-mary hyaloidal system (PHS) (Bergmeister papilla, Mittendorf spot) led to the hypothesis that the cyst originated from the PHS and should therefore be classified as a choristoma [4]. Orellana et al., on the other hand, suggested that the cysts originated from the pigment epithelium, and found it highly unlikely that they were a developmental anomaly [3]. The clinical and histological resemblance to free floating cysts of the iris in the anterior segment supports their possible pigment epithelial origin [9].

Most reports in literature concern pigmented cysts and therefore resemble the first case. Similarly to our study, the reported histopathologic analyses all mention a single layer of cuboidal cells with variable amounts of pigmentation [2], [3], [4], [6]. Some, however, also noted areas of stratification or epithelial fronds [2], [3]. Nork et al. [4] reported a PAS-positive basement membrane, which was also present in this case. Since no electron microscopy was performed, the presence of premelanosomes could not be assessed. The positive staining with AE1/AE3 implies an epithelial origin, which would be expected in either of the previously mentioned hypotheses about the cysts' origin. Negative staining with Mib1 proves that the recorded cyst growth is not related to cell division, but is likely caused by active fluid pumping of the cyst wall [4]. Currently, there is only one histological report of a primary non-pigmented vitreous cyst, which described a multilayer cellular cyst wall and demonstrated neural and glial elements based on immunohistochemistry [5].

These two cases show very distinct clinical characteristics, probably reflecting their distinct origin. In our opinion, both the pigment epithelium and the PHS can give rise to vitreal cysts, but with a different clinical aspect. When cells from the pigment epithelium dislodge during embryogenesis, they form a pigmented cyst, as in the first case. Since the cysts are partially pigmented, Lally et al. [9] argued they originate from the iridociliary sulcus. In that case these cells would have to travel against the aqueous flow and across the zonular fibers to reach the vitreous cavity. It would thus be more likely that the cyst originated from the pars plana.

When the PHS fails to regress, cysts are located in the region of Cloquet’s channel and often other PHS remnants are found [4]. Since the PHS does not contain pigmented cells, the cysts are grey and have a pearly aspect [4]. This matches the characteristics of the second case. One might therefore argue that this type of cyst could be classified as a variant of persistent fetal vasculature.

The presence of high anisometropia in the second case further supports that this cyst is a development anomaly. To our knowledge, this is a new association. Taranath et al. [10] reported a high anisometropia, however the effect of previous cryotherapy should be taken into account in that case. Amer et al. [11], on the other hand, described anisometropia, but with the emmetropic eye containing a cyst.

No specific treatment is necessary for primary vitreous cysts. In the first case, however, surgical removal was preferred, due to the visual disturbances and increasing size. The enlargement of the cyst can be caused by active fluid pumping [4]. In the second case, refractive correction and amblyopia management will be the mainstay of the treatment since there is no obstruction of the visual axis.

## Conclusion

Vitreous cysts are a rare finding with an uncertain pathophysiology. The two cases demonstrate the variety in clinical aspect of these cysts. This likely reflects their origin from either the primary hyloidal system or the pigment epithelium. Specific treatment is often not required.

## Notes

### Competing interests

The authors declare that they have no competing interests.

### Literature search

Pubmed was searched on August 17, 2019, without date restriction for English-language publications containing the search term: vitreous cyst. Only articles concerning primary cysts were discussed.
